# Hair cortisol as psychotherapy process parameter – an inpatient pediatric psychosomatic study

**DOI:** 10.3389/fpsyt.2026.1867478

**Published:** 2026-07-07

**Authors:** Tim Botschek, Anna Röhlich, Burkhard Brosig

**Affiliations:** 1 Family Psychosomatics, Department of Pediatrics and Neonatology, Justus-Liebig University Giessen, Giessen, Germany; 2 Psychoneuroimmunology Laboratory, Department of Psychosomatic Medicine and Psychotherapy, Justus-Liebig University Giessen, Giessen, Germany

**Keywords:** biomarker, hair cortisol concentration, inpatient therapy, longitudinal study, pediatric psychosomatics

## Abstract

**Introduction:**

Pediatric-psychosomatic inpatient therapy is an essential part of the German health care system for the treatment of mental disorders in children and adolescents. However, empirical research in this field remains scarce and limited to psychological parameters. This longitudinal naturalistic study aimed to evaluate the efficacy and sustainability of inpatient psychosomatic therapy in children and adolescents by examining both psychological outcomes and biological markers.

**Methods:**

A total of 58 patients were assessed at seven time points before, during, and after treatment. Hair cortisol concentration (HCC) was measured as a neuroendocrine parameter of long-term stress regulation. Psychometric data were collected using five validated questionnaires.

**Results:**

Findings indicated significant improvements in perceived stress, depressive and anxiety symptoms, family functioning and internalizing symptoms in the course of inpatient treatment. Overall, these effects remained stable at three- and six-month follow-ups, with only transient increases in depressive symptoms and family problems. HCC showed a significant decrease from admission to discharge and remained stable across follow-ups.

**Discussion:**

These results support the efficacy of inpatient pediatric psychosomatic interventions on both psychological outcomes and neuroendocrine stress regulation and highlight the value of integrating biological markers into psychotherapy research.

## 1 Introduction

The mental health of children and adolescents has gained increased attention in recent years, with numerous studies reporting a rise in psychosocial distress in this age group (1–4). As a result, both outpatient and inpatient treatment demands have grown substantially (5–7).

The concept of pediatric psychosomatics has become particularly relevant in the inpatient treatment of children and adolescents with mental disorders. It encompasses the psychological treatment of children and adolescents in a pediatric hospital setting. Children and adolescents with treatment needs specific to an inpatient hospital setting are admitted. Indications included, in particular, distinguishing between functional symptoms and organic disorders, as well as promoting compliance and coping with illness. Within the framework of specific pediatric psychosomatic inpatient treatment, there are clear guidelines regarding interdisciplinary, multidimensional therapy, which are codified in German social law with clear structural and time requirements (8, 9).

In recent years, pediatric psychosomatics has become a vital part of the German healthcare system. Fundamental to the clinical concept of pediatric psychosomatic care is the assumption that psychosomatic disorders can only be comprehensively understood if biographical, social, mental and biological aspects are taken into consideration simultaneously. The corresponding multimodal treatment approach therefore integrates psychotherapeutic, somatic and family-related dimensions of illness and recovery (8). In extension of psychiatric concepts, its multidimensional approach, focus on the treatment of chronic somatic illnesses, and strong therapeutic focus represent a significant addition to basic pediatric psychotherapeutic care.

Despite its clinical relevance, empirical research on pediatric psychosomatic therapy remains limited and does not reflect the complexity of care concepts offered in the clinical field. There are studies on the effectiveness of psychosomatic therapy in adults (10, 11), but only a few studies that have subjected inpatient pediatric psychosomatic therapy to empirical testing at all. However, there is preliminary evidence of the effectiveness of such treatment (12).

Most existing studies rely on simple pre-post designs and rarely include follow-up assessments. Consequently, long-term treatment effects remain largely unexplored, although follow-up data are essential to distinguish sustained improvements from temporary symptom relief or diagnostic shifts (13–15).

In addition to a lack of follow-up data, the existing literature in the field of pediatric psychosomatics does not adequately reflect the complexity and multi-faceted nature of the treatment modality and its outcome. Prior research has predominantly focused on psychological outcomes assessed via self-report questionnaires, which can be prone to bias (16). The narrow focus on psychological data clearly neglects the biopsychosocial complexity of psychosomatic disorders by omitting social and especially biological factors. Moreover, Mayeux (17) emphasizes that, in contrast to self-reports, biomarkers such as cortisol provide more reliable, valid and accurate data.

Hair cortisol concentration (HCC) has emerged as a promising cumulative indicator of long-term stress exposure (18, 19). The relevance of HCC in the field of somatic diseases such as Cushing-syndrome is well documented [e.g (20).]. Lately, altered HCC levels have also been reported in several mental health conditions (21) and together with additional stress markers (22). Thus, the presence of mental distress and illness appears to be associated with changes in HCC, although the direction of this effect remains unclear. For some populations, such as children and adolescents with victimization, mood dysregulation and externalizing disorders, higher HCC levels were found (23–25). Other authors reported lower HCC in children and adolescents with depression or anorexia nervosa [26, 27; for a detailed overview for adults see (21)].

A Canadian study found three different trajectories of HCC in children and adolescents with chronic physical illnesses. Persistently elevated HCC levels were associated with increased psychopathological symptoms, whereas declining HCC trajectories were associated with improved mental health (28).

Due to the change in HCC in the context of mental disorders, HCC has also been increasingly discussed as a biomarker for tracing psychotherapeutic processes. An overview of the topic is provided by Botschek and colleagues (29). The authors were able to identify around a dozen studies that used HCC as a psychotherapeutic process parameter. The available studies did not yet appear to show a consistent picture of the course of HCC with psychotherapeutic interventions, although a trend towards a reduction in HCC was observed in the course of such treatments.

To date, no study has investigated HCC as a psychotherapeutic process parameter within the inpatient pediatric psychosomatic treatment. This study therefore aims to fill this research gap by assessing both psychosocial and biological outcomes in a longitudinal naturalistic design that includes two follow-up assessments, therefore representing a significant extension to existing research. Specifically, the following hypotheses are to be answered:

1. Inpatient pediatric-psychosomatic multidimensional therapy leads to a significant reduction in perceived stress, depressive and anxious symptoms, internalizing symptoms and family problems.
2. These effects are sustainable and persist three and six months respectively after discharge.
3. Inpatient treatment is also associated with the reduction of biological stress markers, operationalized via HCC, and this effect is maintained at the three- and six-month follow-up assessments.

## 2 Materials and methods

### 2.1 Study design and implementation

The study was conducted at the Department of Family Psychosomatics at Giessen University Hospital, Germany, from February 2022 to February 2024. Admission to inpatient treatment is based on several outpatient interviews, at the end of which there is a visit to the ward and an interview with the ward’s nursing staff. The first time point takes place during the nursing interview.

**Figure 1:**
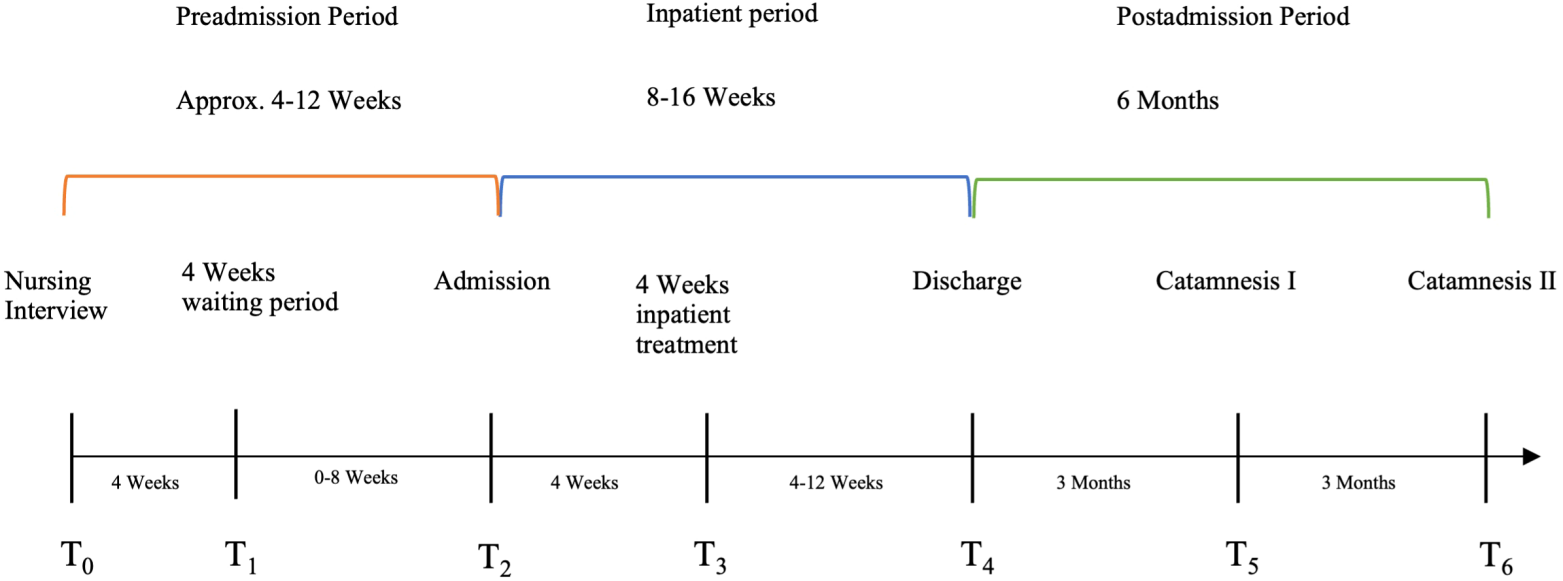
Study design.

The study design includes a total of 7 measurement points (T_0_ – T_6_; see **Figure 1**). After the first time point described above (T_0_), a second measurement (T_1_) takes place 4 weeks after the first one. T_2_ represents the inpatient admission, the mean duration between T_2_ and T_1_ is 28 days (SD = 40.47). T_3_ is carried out 4 weeks after admission and T_4_ on discharge. The time between T_4_ and T_3_ averages 65 days (SD = 33.48). T_5_ and T_6_ are two follow-up time points three and six months after discharge respectively. The study design was originally conceptualized to allow for an intra-individual comparison of patients during the waiting period (T_0_–T_1_) versus the initial treatment period (T_2_–T_3_), each lasting 4 weeks, in the sense of a waiting-list control group. However, it became apparent that, given the reality of care with a very high volume of acute admission requests, the rigor of these design requirements was not clinically or ethically justifiable.

At each time point, standardized psychometric questionnaires were administered and hair samples were collected by trained psychologists.

The study was approved by the local ethics committee of the Department of Medicine at Justus-Liebig-University, Giessen and was registered on the German Clinical Trials Register (DRKS00033917).

### 2.2 Sample characteristics

All patients who were admitted to the Family Psychosomatics unit during the specified period and were seeking inpatient treatment were invited to take part in the study. The indication for inpatient treatment is often made in cases of school absenteeism, significant symptom burden and, especially, the failure of outpatient therapy attempts. None of the participants had known endocrine disorders affecting cortisol secretion or received systemic corticosteroid treatment during the study period or the corresponding HCC assessment window.

In total, only 3 patients declined to participate. Of the 58 subjects included, 69% were female (*n* = 40) and 31% male (*n* = 18) with a mean age of 15.24 years (*SD* = 1.97, range: 10 – 18).

Treatment duration ranged from 10 to 155 days (*M* = 85.77 *SD* = 38.62). Patients who discontinued treatment prematurely were retained in the analysis to preserve the naturalistic character of the design.

Diagnostic categories can be divided into five groups, as shown in **Table 1**. Mainly mood disorders such as depression or dysthymia, neurotic and somatoform disorders such as somatization or conversion disorders as well as behavioral syndromes associated with physical factors such as anorexia nervosa or problems with the acceptance and compliance of somatic disorders were treated.

**Table 1:** Treated disorders coded according to ICD-10.

Diagnosis	N	%
F3 Mood (affective) disorders	15	26
F4 Neurotic, stress-related and somatoform disorders	26	45
F5 Behavioural syndromes associated with physiological disturbances and physical factors	11	19
F6 Disorders of personality and behaviour	1	2
F9 Behavioural and emotional disorders with onset usually occurring in childhood and adolescence	5	9

The diagnosis was made with the help of the Diagnostic Interview for mental disorders in children and adolescents (Kinder-DIPS (30)), a structured clinical interview that is well-validated and commonly used in German-speaking countries (31).

For reasons discussed below, only 10 of the participants completed the full number of time points. Clinical considerations took always precedence over scientific ones. **Table 2** displays the exact number of participants for every time point.

**Table 2:** Number of participants for each time point.

Time point	N
0	44
1	24
2	48
3	39
4	41
5	28
6	20

### 2.3 Intervention

The participants were treated in the Family Psychosomatics unit of the University Hospital in Giessen, Germany, which is part of the Department of Pediatrics and Neonatology. The ward has been in existence since 2017 and offers 10 treatment places. Therapeutically, treatment follows the psychoanalytic and family-dynamic approach initially described by Horst-Eberhard-Richter (32, 33; see also 8, 12).

The treatment program is multidimensional. There is a strong focus on the psychotherapeutic processes and its modulation by appropriate therapeutic interventions. All patients have individual therapy twice a week and family therapy once a week. In addition, they participate in group therapy four times a week, in educational groups and in physiotherapy and a variety of creative therapies such as art therapy and music therapy (a total of about 12 sessions per week). These treatment modules are carried in the whole group of patients present on the ward (maximally 10) and all patients participate in the same modalities.

The psychotherapeutic team consists of medical doctors, psychologists, physiotherapists, creative therapists and other educators. The multi-professional team is complemented by a specialized nursing staff, who are essential for structuring everyday activities and communication.

### 2.4 Measures

#### 2.4.1 Clinical variables

##### 2.4.1.1 Perceived stress questionnaire

The PSQ (34) records the current, subjectively perceived stress level using 30 items. The internal consistency was in the good range with values of.80 -.86.

##### 2.4.1.2 Beck depression inventory for youth

The BDI-Y is part of the Beck Youth Inventories – Second Edition (35). Using 20 items, depressive symptoms such as dejection, negative assumptions about oneself and physiological symptoms of depression are assessed. The German version of the BDI demonstrated excellent internal consistency scores (Cronbach’s Alpha.90 -.94).

##### 2.4.1.3 Beck anxiety inventory for youth

Just as the BDI-Y, BAI-Y is part of the Beck Youth Inventories – Second edition (35) and consists of 20 items. It includes questions about specific and generalized anxieties and specific symptoms of anxiety such as rumination. The German version revealed good to excellent internal consistency scores (Cronbach’s Alpha.86 -.92).

##### 2.4.1.4 Youth self-report

The YSR is part of the Child Behavior Checklist (36). Using 112 items, it evaluates conduct and emotional problems, somatic symptoms and social competencies in adolescents. Eight subscales and two superordinate scales, internalizing and externalizing problems, can be generated, as well as an overall problem scale. The scale of internalizing problems is of special interest in the context of psychosomatic disorders (37). Therefore, the statistical analysis will focus on this scale. With a Cronbach’s alpha score of.89, the internal consistency is in the good range.

##### 2.4.1.5 Allgemeiner familienbogen (general family questionnaire)

The FB is part of Cierpka and Frevert’s family questionnaires (38). In 40 items, perceived family problems in seven different areas are queried and family coherence is assessed. An overall scale can be created from the seven subscales. The internal consistency of the scales is low but still acceptable with Cronbach’s Alpha between.51 and.75.

#### 2.4.2 Biological variables

Representative for biological effects of distress, HCC was determined. To measure the HCC, 3 cm long hair samples were taken from the posterior vertex as close to the scalp as possible according to the standard operating procedure protocol provided by the Psychoneuroimmunology (PNI) Laboratory at the Department of Psychosomatic Medicine of the Justus-Liebig-University, Gießen. Sampling psychologists were trained by the PNI team. Samples were stored at room temperature in a dark container until analysis at the laboratory.

The average hair growth is 1 cm per month. The analysis of cortisol levels in the 3 centimeters closest to the scalp represents the cumulative production over the last 3 months (39). The determination of HCC in the samples was achieved by following a protocol established in the PNI laboratory (22). In brief, this includes the pulverization with the aid of a ball mill followed by weight-adapted methanol-cortisol extraction. Lyophilized samples were stored at -80°C until bulk analysis. For the quantification, a commercially available Enzyme-linked Immunosorbent Assay (ELISA) was used. According to the manufacturer’s specifications, the intra- and inter-assay coefficients of variation are +4.3% and +13.2%, the sensitivity of cortisol ELISA is 0.005 µg/dl (standard range 0.15–30 ng/ml). In accordance with this protocol, results are reported as µg/dl of suspended hair extract, reflecting the concentration measured in the resuspended extract obtained from pulverized hair. Values that were detectable but fell below the lower limit of quantification (LLOQ) were treated as left-censored. In Line with common clinical practice for biomarker data, these values were imputed as LLOQ/2 (see also (40). The proportion of imputed values was low (7%). However, we additionally ran sensitivity analyses excluding these values, which yielded a similar pattern of results. For a more detailed description of the procedure see (41) and (42).

### 2.5 Statistical analysis

Given the longitudinal design with repeated measures and the presence of missing data at various time points, linear mixed models (LMMs) were selected as the primary statistical method (43, 44). A random intercept for each participant was included to account for within-subject correlations across time points. Time point was treated as a fixed factor. A first order autoregressive covariance structure (AR1) was specified to model decreasing correlations among repeated measures across adjacent time point. The model was estimated using the Restricted Maximum Likelihood method (REML). This analytical approach ensures that both the hierarchical structure of the data (observations nested within individuals) and the temporal autocorrelation of repeated measurements are appropriately modeled, increasing the robustness and interpretability of the statistical findings. HCC analyses were adjusted for age, sex and body mass index (BMI) in accordance with the recommendations of previous research regarding the selection of covariates [see, for example (19, 45)].

To further investigate specific hypotheses regarding differences between particular time points, custom contrasts were defined and tested within the model. For the contrast comparing T_2_ (admission) and T_4_ (discharge), we formulated a directional *a priori* hypothesis, expecting a decrease in symptom burden and HCC from admission to discharge.

In contrast, the comparisons of T_4_ (discharge) with T_5_ (3-month follow-up) and T_6_ (6-month follow-up) were non-directional, as no systematic change was expected across these follow-up intervals. In Addition, effect sizes were calculated. Pearson correlations were computed to illustrate the association between psychometrics and HCC for each time point.

The statistical analyses were conducted using IBM SPSS Version 23 (46).

## 3 Results

### 3.1 Psychometric variables

Descriptive statistics for all outcome variables across time points suggested a decrease of symptom severity from admission (T_2_) to discharge (T_4_) (**Table 3**). Compared to T_2_, this decrease in mean scores remained stable across follow-ups (T_5_ and T_6_). Descriptively, the highest patient-reported outcomes were observed at T_1_ prior to admission. In addition, inspection of the mean scores suggested a mild increase in most outcome measures (except anxiety) between discharge (T_4_) and the three-month follow up (T_5_), followed by a decline towards discharge levels at six-month follow up (T_6_).

**Table 3:** Mean values and standard deviation for the psychometric outcomes per time point.

Time point	T0	T1	T2	T3	T4	T5	T6
Parameter							
PSQ	0.58 (0.21)	0.63 (0.20)	0.53 (0.21)	0.58 (0.22)	0.48 (0.24)	0.53 (0.24)	0.44 (0.25)
BDI	25.70 (12.30)	28.55 (13.69)	24.82 (14.36)	24.23 (14.41)	17.97 (12.04)	21.52 (12.99)	18.05 (12.53)
BAI	23.41 (12.50)	28.05 (12.50)	22.77 (13.55)	23.33 (14.95)	17.58 (11.95)	19.70 (13.46)	19.65 (13.55)
YSR	28.55 (12.80)	30.86 (13.90)	26.49 (12.54)	27.21 (13.17)	23.33 (13.75)	25.81 (13.95)	22.40 (14.86)
FB	32.89 (16.02)	39.55 (13.43)	35.27 (17.43)	34.23 (17.66)	31.50 (16.30)	36.23 (16.08)	29.50 (14.31)

PSQ, Perceived Stress Questionaire; BDI, Beck Depression Inventory for Youth; BAI, Beck Anxiety Inventory for Youth; YSR, Internalizing scale of the Youth Self Report; FB, Allgemeiner Familienbogen (General Family Questionaire). Values are presented as mean (SD).

The analysis of the linear mixed models is illustrated in detail in **Table 4**. For the perceived stress load, the linear mixed model also showed a significant main effect of time for the PSQ, *F*(6, 114.34) = 5.16, *p* <.001. The planned contrasts between admission and discharge showed a significant decrease in stress load (*p* = .015, *d* = 0.22). The perceived stress level remained stable three months (*p* = .410, *d* = 0.21) and six months (*p* = .113, *d* = 0.16) after discharge.

**Table 4:** Contrast analyses for psychometric data.

Contrast	PSQ (*MD*, (95% CI))	*p*	BDI (*MD*, (95% CI))	*p*	BAI (*MD*, (95% CI))	*p*	YSR (*MD*, (95% CI))	*p*	FB (*MD* (95% CI))	*p*
T2 vs. T4	0.06, (0.01, 0.11)	.030^*^	7.73 (4.50, 10.94)	<.001^***^	5.79 (2.96, 8.63)	<.001^***^	3.38, (0.34, 6.42)	.029^*^	4.73 (1.72, 7.75)	.002^**^
T4 vs. T5	-0.02, (-0.07, 0.03)	.410	–3.22 (–6.18, –0.25)	.034^*^	–1.33 (–4.13, 1.47)	.349	-0.92, (-3.74, 1.90)	.519	–3.36 (–6.39, 0.33)	.030^*^
T4 vs. T6	0.06, (-0.01, 0.12)	.113	–0.27 (–4.47, 3.94)	.901	–1.68 (–5.41, 2.05)	.375	2.44, (-1.58, 6.45)	.233	–0.90 (–4.87, 3.07)	.655

MD, Mean Difference; 95% CI, 95% Confidence Interval. ***p <.001, **p <.005, *p <.05.

Concerning the BDI, there was a significant main effect of time, *F*(6, 120.80) = 5.48, *p* <.001. The planned contrasts analyses revealed significantly lower depression scores at discharge compared to admission (*p* <.001, *d* = 0.51), indicating a significant treatment effect. However, there was a significant increase in depressive symptoms from discharge to the three-month follow-up (*p* = .034, *d* = 0.29), followed by an improvement at the six-month follow-up, with no significant differences compared to discharge (*p* = .901, *d* = 0.01).

The BAI also showed a significant main effect of time, *F*(6, 127.24) = 4.09, *p* <.001. The planned contrasts showed a significant decline in anxious symptoms between admission and discharge (*p* <.001, *d* = 0.40). These effects remained stable three (*p* = .349, *d* = 0.17) and six month (*p* = .375, *d* = 0.17) post-treatment.

The analysis of the General Family Questionnaire revealed a significant main effect of time, *F*(6, 118.13 = 2.96, *p* = .010). Contrast analyses showed a significant improvement in family functioning at discharge compared to admission, *p* = .001, *d* = 0.22. The perceived family problems increased significantly three months after discharge (*p* = .030, *d* = 0.29). At six months follow-up, however, there were no differences in family functioning, compared to discharge (*p* = .655, *d* = 0.13).

The evaluation of the internalizing scale of the YSR showed a significant effect of time, *F*(6, 123.34) = 2.34, *p* = .036. The planned contrasts indicated a significant reduction in internalizing symptoms in the course of treatment (*p* = .015, *d* = 0.24). No significant changes in internalizing symptoms emerged between discharge and three (*p* = .519, *d* = 0.18) and six-month (*p* = .233, *d* = 0.07) follow-up.

### 3.2 Hair cortisol concentration

Descriptively, the highest HCC was observed at admission and not prior to admission, as was the case for the patient-reported outcomes (**Table 5**). Similarly, however, the mean HCC appeared higher at admission (T_2_) than discharge (T_4_) and suggested a temporary increase at three-month follow-up followed by a decline at six-month follow up (T_6_).

**Table 5:** Mean values and standard deviation for HCC per time point, in µg/dl suspended hair extract.

Time point	T0	T1	T2	T3	T4	T5	T6
Parameter							
HCC	0.023 (0.042)	0.018 (0.019)	0.059 (0.170)	0.029 (0.029)	0.024 (0.030)	0.039 (0.132)	0.022 (0.021)

Values are presented as mean (SD).

Looking at the Linear Mixed Models, there was a non-significant main effect of time for HCC after controlling for age, sex and BMI (*F* (6, 118,971) = 0.90, *p* = .495). Parallel to the patient-reported outcome data however, the planned contrasts revealed a significant decrease in HCC between admission (T_2_) and discharge (T_4_) (*p* = .038, *d* = 0.39). There were no significant changes in HCC three (*p* = .492, *d* = 0.16) and six months (*p* = .824, *d* = 0.07) post treatment, compared to discharge.

### 3.3 Correlation analyses

Correlation analyses were conducted to examine the associations between HCC and psychometric variables for each time point. Only one significant correlation occurred: For T_2_, the association between HCC and PSQ was significant, *r* = .293, *p* = .048.

## 4 Discussion

This study provides evidence for the effectiveness and sustainability of inpatient pediatric-psychosomatic multidimensional therapy by analyzing patient-reported outcomes indicating distress and mental health as well as the biological dimension. For the first time in this context, HCC is reported together with mental health indicators as a potential process-related biomarker in a population of pediatric psychosomatic patients. In the course of the treatment, patients showed significant reductions in stress related, depressive, anxious, internalizing symptoms, accompanied by improved family functioning. Overall, these improvements of patient-reported outcome measure results were maintained at three- and six-month follow-ups, supporting the lasting clinical relevance of this treatment. HCC showed a comparable trajectory with a significant decrease from admission to discharge, adjusted for age, sex and BMI, suggesting that inpatient child psychosomatic treatment has demonstrable effects not only on the psychosocial, but also on the biological level.

The significant reduction of psychosocial symptom burden aligns with the few previous findings that also evaluated the effects of inpatient pediatric psychosomatic therapy [e.g (12).]. However, the present study represents a significant extension of previous research. In contrast to the existing literature, the present study explicitly recorded symptoms of depression and anxiety, not only broader mental burden. This approach revealed their significant reduction in the course of treatment. Patients submitted to inpatient care had not sufficiently benefited from outpatient treatment prior to admission. Their improvement during multimodal psychosomatic inpatient care highlights the added value of an intensive, structured inpatient environment. The patients studied here presented with complex issues that included psychological, somatic, psychosocial and family aspects, representative for patients in inpatient care. Their complexity appeared to exceed the scope of outpatient settings. The intensive therapeutic milieu, which provides for a variety of psychotherapeutic approaches and complementary forms of therapy, seemed to be beneficial for these patients [see also (47)]. In addition, the adolescents may have profited from the educational framework provided and from the intensive contact with their peers. These aspects of the inpatient care foster accelerated socialization and the activating, lively environment counteracts social withdrawal and passivity, thereby reducing depressive, anxious and broader internalizing symptoms.

The temporary increase in depressive symptoms at the three-month follow-up may reflect transitional challenges during re-integration in everyday life. Particularly increased at this time point are perceived family problems. Patients move from a structured and emotionally supportive setting, which counteracts depressive tendencies, back into more demanding family and school contexts, which may temporarily increase symptom expression. The family problems reported as intensified three months after discharge may also reflect distress and conflicts during the detachment process possibly initiated during the treatment. These changes in family structures, including the reorganizing of family roles, initially pose a challenge to the family structure. However, family tension appears to calms down after six months, indicating a new familiar relatedness and healing. The stabilization at six-month follow-up suggests a successful adaptation processes and ultimate consolidation of treatment gains.

Compared with the admission, the patients reported fewer family problems at discharge. Therapeutically, the patients are often seen as index patients of a pathological family system in the sense of Horst-Eberhard Richter (32, 33). The mental disorders of adolescents signal existing family problems (48). The significant improvement in family functionality is therefore of particular importance and even a necessary condition in the long-term healing of patients (49). From the perspective of family dynamics, such an improvement is only possible by gradually working through family conflicts and restoring emotional relatedness within the family. The temporary separation of the adolescent from his parents seems to create the necessary distance to be able to discuss conflictual issues with the family in a protected environment [see also (50)]. If the patients are released into a healthy, supportive family environment, the long-term prognosis appears promising. The lasting effects also found in the follow-up examinations after six months underline this assumption.

Reductions in perceived stress support findings from previous literature, which link psychotherapy to stress alleviation (51). Stress is a transdiagnostic factor contributing to a range of psychological and psychosomatic symptoms (52, 53). Its reduction may therefore mediate broader improvements in well-being. Stress as a basal marker of suffering is still significantly reduced six months after discharge, underlining the far-reaching and fundamental influence of psychosomatic treatment also on the patient’s physical well-being.

A major contribution of this study is the integration of HCC as a cumulative biological stress marker. The significant increase in HCC in the three months prior to admission suggests that submission to the ward as an acute care clinic occurs in a situation of great need. Patients appear to have accumulated significant stress-levels upon admission, which can be traced in their biological stress levels covering the three months prior to admission. The significant decline of HCC during inpatient treatment supports the hypothesis that psychological improvement is accompanied by measurable physiological changes and aligns with growing evidence that psychotherapeutic interventions can modulate neuroendocrine stress systems (21, 29). In the context of pediatric psychosomatic therapy, this finding is particularly relevant. In psychosomatic disorders, internal or family conflicts manifest themselves not only on a psychological, but also on a somatic level. The dysregulation of the HPA axis is discussed as a key mechanism that may explain the manifestation of psychosomatic symptoms in response to these kinds of distress (54, 55). A preferably long-term stabilization of the neurobiological system governing cortisol release, as indicated by a reduction in HCC, could thereby contribute in particular to improving the well-being of patients. The observed decrease in HCC may suggest a treatment-associated modulation of HPA-axis activity, although the clinical meaning of this change remains preliminary given the heterogeneity of the sample and the lack of established pediatric reference values. These changes and the alleviation of physical stress processes appear to be stable at least across the follow-up period.

The absence of consistent correlations between HCC and psychological measures were reported also by other authors, which found higher correlations between HCC and other somatic stress indices than with perceived stress [e.g (56, 57).]. It can be explained in part by methodological factors, as HCC represents a cumulative long-term parameter, while psychometric instruments capture current internal states. Moreover, many psychosomatic patients experience high levels of alexithymia, which means they are emotionally insulated or neutralized, with little access to their own inner experiences (58). This inner barrenness makes it considerably more difficult for them to describe their own emotional state in self-reports. In this sense, the lack of correlations between psychological and somatic stress is consistent. Other authors also refer to a psyche-soma disconnection in the context of psychosomatic disorders, which can also be demonstrated empirically by the lack of significant correlations (59). This makes the reduction in somatic stress in the form of HCC all the more significant as an indicator of an objectively measurable change in well-being.

Besides the mentioned strengths, some limitations warrant consideration. The present study lacks an untreated control group, which would allow the effects of the inpatient therapy to be distinguished from randomly occurring remission effects. Future research should therefore consider the inclusion of a control group to enable a differentiated interpretation of the effects found. With a total of 58 participants, an acceptable number of patients took part in the study. However, the number of complete datasets was small, mainly due to clinical reasons like urgent admissions or early discharges, as well as limited follow-up participation. To preserve the naturalistic character of the study, these subjects were also included in the statistical evaluation. The analysis based on a data set of this kind therefore underlines its practical relevance due to its proximity to clinical routines. However, larger sample sizes could increase statistical power and facilitate more specific analyses of individual groups, e.g. divided by diagnostic category or age. This is particularly relevant because the disorders treated, although all subsumed under the umbrella term of psychosomatic disorders, must be considered heterogeneous and their interrelationships with biological processes vary, considering for example anorexic disorders. In particular, the interpretation of the results of the follow-up examinations must remain preliminary due to the limited number of participants and should be expanded upon in future studies, both in terms of the number of participants and the follow-up period.

Statistically, this unbalanced data set was addressed by using a Linear Mixed Model. Compared to an ANOVA with repeated measures, the LMM offers a much more adaptable way of dealing with unbalanced experimental designs and missing data sets (60). The fact that unbalanced experimental designs do not necessarily mean a loss of statistical power, especially in psychotherapy research, was emphasized by Hsu (61), among others.

Concerning the determination of HCC, several methodological issues are to be taken into account when interpreting the results. Accepted reference values for HCC in youths are not yet available, even though attempts to establish these standards are already being made [e.g (62).]. The question of whether the HCC values found here are expected or in particular conspicuous can therefore only be answered preliminarily. In addition, the methods of collecting HCC differ between studies, although they correlate highly with each other and a consistent trend towards higher HCC values was reported in immunoassay compared to mass-spectrometry (63). Additionally, HCC values also vary depending on the investigating laboratory, which limits the generalizability of the effects found (45). Existing studies have shown that variables such as BMI or gender have a significant influence on HCC, although the gender differences appear to level out during puberty (64). The mentioned study also highlighted the importance of pubertal stage in interpretating HCC values. Pubertal stages were not systematically determined and controlled for in this study. Given the age range of the sample, differences in pubertal maturation may have contributed to variability in HCC levels. Finally, evaluations divided into subgroups, also with regard to diagnoses, could therefore provide additional information. For example, a study by Nyengaard and colleagues (65) found lower HCC values in patients with functional somatic complaints, while depressive symptoms may be related to the high and low end of HCC (66). A limiting factor is therefore the considerable variation in HCC values. However, high variance is consistent with the existing literature on HCC and represents, not least, its character as a cumulative long-term marker of stress and high interpersonal variance of stress experience and levels. In control of this limitation, analyses excluding outliers (>3 SD) did not reveal any relevant changes in the results.

### 4.1 Conclusion

In conclusion, this study provides convincing evidence that multidimensional inpatient pediatric-psychosomatic therapy is associated with improvements in psychological outcomes and changes in neuroendocrine stress regulation. Patient-reported improvements were largely maintained across the six-month follow-up period, while HCC decreased from admission to discharge and remained stable thereafter, underscoring the impact of this clinical approach on a psychological and neuroendocrine level. The integration of biological markers such as HCC enriches the understanding of psycho*somatic* processes and highlights the importance of biopsychosocial research in pediatric mental healthcare.

## Data Availability

The raw data supporting the conclusions of this article will be made available by the authors, without undue reservation.
